# Dietary *E. coli* promotes age-dependent chemotaxis decline in *C. elegans*

**DOI:** 10.1038/s41598-024-52272-4

**Published:** 2024-03-06

**Authors:** Nadia Suryawinata, Rikuou Yokosawa, Ke Hui Cassandra Tan, Alison Lok Lai, Ryusei Sone, Ikue Mori, Kentaro Noma

**Affiliations:** 1 Group of Nutritional Neuroscience, Graduate School of Science, Neuroscience Institute, Nagoya University, Nagoya, 464-8602 Japan; 2 Group of Microbial Motility, Division of Natural Science, Department of Biological Science, Graduate School of Science, Nagoya University, Nagoya, 464-8602 Japan; 3 Group of Molecular Neurobiology, Graduate School of Science, Neuroscience Institute, Nagoya University, Nagoya, 464-8602 Japan; 4 Present Address: Division of Biology and Biological Engineering, California Institute of Technology, 1200 E California Blvd, Pasadena, CA 91125 USA

**Keywords:** Senescence, Behavioural genetics, Gene expression, Bacteria, Neural ageing, Transcription

## Abstract

An animal’s ability to sense odors declines during aging, and its olfactory drive is tuned by internal states such as satiety. However, whether internal states modulate an age-dependent decline in odor sensation is unknown. To address this issue, we utilized the nematode *Caenorhabditis* *elegans* and compared their chemotaxis abilities toward attractive odorants when aged under different dietary conditions. Feeding with the standard laboratory diet, *Escherichia coli* attenuated the chemotaxis ability toward diacetyl, isoamyl alcohol, and benzaldehyde when aged. On the other hand, feeding with either the lactic acid bacteria *Lactobacillus reuteri* or food deprivation selectively maintained the chemotaxis ability toward diacetyl. Our results suggest that ingestion of *E. coli* causes age-dependent chemotaxis decline. The changes in the chemotaxis behavior are attributed to the different expressions of diacetyl receptor *odr-10*, and the chemotaxis behavior of aged animals under food deprivation is shown to be dependent on *daf-16*. Our study demonstrates the molecular mechanism of how diet shapes the trajectory of age-dependent decline in chemosensory behaviors.

## Introduction

Our ability to discriminate odors declines at a certain age^1^. While cellular mechanisms of age-dependent olfactory decline, such as loss of selectivity of olfactory neurons, are proposed^2^, the molecular mechanism remains to be elucidated. Moreover, how diet modulates age-dependent sensory decline is largely unknown^3^.

The nematode *Caenorhabditis* *elegans* is ideal for studying the molecular mechanisms of age-related changes in the nervous system due to its relatively short lifespan, simple nervous system, and genetic tractability^4^. To study the age-dependent decline of odor sensation in *C. elegans*, we used chemotaxis, the innate attraction toward volatile odorants, such as diacetyl, benzaldehyde, and isoamyl alcohol^5–7^. These odorants are often found as byproducts of fermentation in bacteria, which are their food source^8^. In *C.* *elegans*, diacetyl chemotaxis of different concentrations involved specific neurons; in young adult animals, the AWA sensory neurons are necessary for the chemotaxis of lower concentrations of diacetyl, whereas both AWA and AWC sensory neurons redundantly function toward detection of higher concentrations^5,9^. Detection of a low concentration of diacetyl (0.1%) requires the G-protein coupled receptor ODR-10 expressed in AWA^10^. On the other hand, the AWC neurons are required during chemotaxis toward isoamyl alcohol and benzaldehyde^5^.

Using chemotaxis as a model, we investigated the dietary modulation of age-dependent decline in odor sensing by comparing three different dietary conditions: *E. coli*, the standard diet for *C. elegans* in the laboratory^11^; *Lactobacillus reuteri,* a lactic acid bacterium that affects age-dependent associative learning behaviors in *C. elegans* without changing their lifespan^12^; and complete deprivation of food. We showed that the chemotaxis response towards low concentrations of volatile odorants was attenuated in aged animals fed with the standard laboratory diet, *E. coli*. On the other hand, both *L. reuteri*-fed and food-deprived aged animals maintained chemotaxis ability, specifically towards the odorant diacetyl. This result indicates that while *E. coli* causes a general effect of age-dependent chemotaxis decline, *L. reuteri* and complete food deprivation mitigates this decline in an odorant-specific manner. The age- and diet-dependent chemotaxis phenotypes are correlated with the mRNA expression of *odr-10*, a gene encoding the diacetyl receptor. The high chemotaxis ability of *L. reuteri-*fed and food-deprived aged animals was dependent on DAF-16, a downstream transcription factor of the insulin signaling pathway, which is known to be involved in the lifespan of aged animals^13,14^ and age-dependent decline of associative learning behavior^12^.

## Results

### Chemotaxis ability declines with age

**Figure 1:**
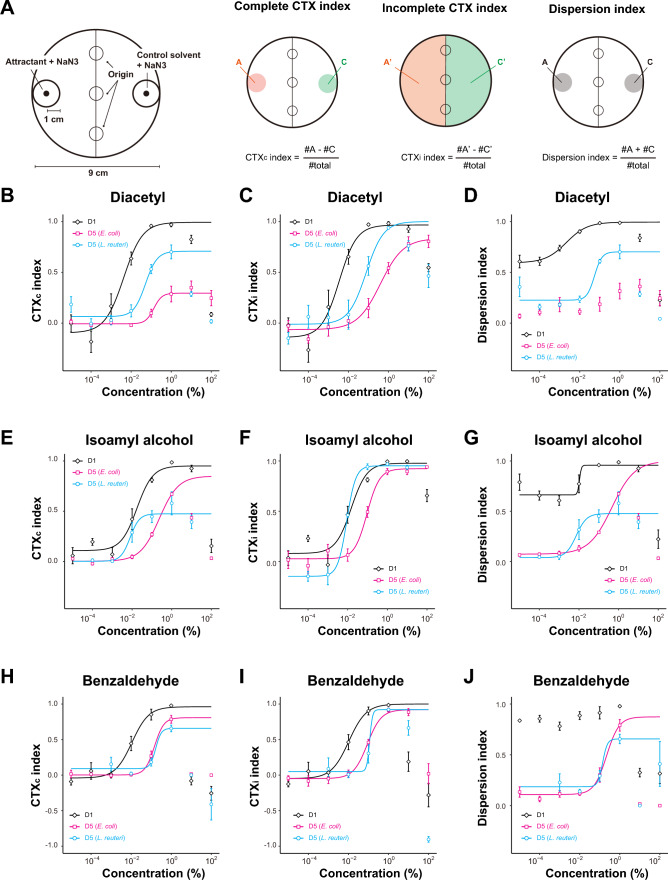
Modulation of chemotaxis abilities by age and diet. (**A**) Schematic representation of a chemotaxis plate and equations for indices. Attractant and control solvent were spotted on opposite ends of the plate. Approximately 100–150 animals were placed on the center of an assay plate and allowed to roam freely for one hour at room temperature. Chemotaxis complete (CTX_c_) and incomplete (CTX_i_) indices reflect animals’ ability to reach the attractant (orange area) and sense the attractant, respectively. The dispersion index reflects animals’ ability to explore on the plate. (**B**–**J**) Chemotaxis dose–response curves of *E. coli*-fed D1, D5 *E. coli*-fed or *L. reuteri*-fed animals toward diacetyl, isoamyl alcohol, and benzaldehyde. Data with significantly lower values than those at the highest lower concentration were excluded from fitting (see Materials and Methods). Error bars indicate S.E.M.

We first characterized the chemotaxis behavior of young adult (day 1 of adulthood, D1) animals cultivated under our laboratory conditions toward three volatile odorants: diacetyl, isoamyl alcohol, and benzaldehyde. To quantitate chemotaxis ability, we performed population chemotaxis assays and defined the following indices (Fig. 1A)^5^: (1) the complete chemotaxis (CTXc) index, which reflects the ability to sense odorants and complete migration toward the odorant source, (2) incomplete chemotaxis (CTXi) index, which reflects the ability to sense odorants and can be high even when animals cannot reach the attractant, (3) the dispersion index, which indicates the number of animals that reached either attractant or control area, and thus reflects the animals’ overall ability to move. We examined chemotaxis behaviors toward three odorants across a wide range of concentrations (10^–5^–10^2^%) and calculated dose–response curves (Fig. 1B–J). The parameters of the dose–response curves are summarized in Supplementary Table 1. As previously shown^5^, young animals showed chemotaxis behavior toward diacetyl (Fig. 1B and C, black) and isoamyl alcohol (Fig. 1E and F, black) in a dose-dependent manner up to 10%; animals were attracted to benzaldehyde up to 1% but were repulsed at 100% (Fig. 1H and I, black). The CTXc and CTXi indices were similar at D1, suggesting that young adult animals can sense and complete the locomotion towards an attractive odorant source. At D1, the dispersion indices were relatively high even at the low concentration of odorants (Fig. 1D, G, and J, black), suggesting that D1 animals are generally exploratory, even when they do not sense any attractants.

We next characterized the effect of aging on the chemotaxis ability. To prepare aged animals, we treated them with 5-fluorodeoxyuridine (FUdR), which prevents their offspring from hatching^15^. When fed with the standard laboratory diet, *E. coli*, aged (day 5 of adulthood, D5) animals showed a rightward shift of the CTXi dose–response curves for all three odorants, suggesting a lower sensitivity towards the odorants tested (Fig. 1C, F, and I, magenta). EC50 values of the CTXi index drastically increased for diacetyl (113.5 times) and only mildly increased for both isoamyl alcohol (8.4 times) and benzaldehyde (6.3 times) (Supplementary Table 1). For both isoamyl alcohol and benzaldehyde, aged animals could still complete chemotaxis toward higher odorant concentrations, suggesting that D5 animals were still motile at this age (Fig. 1E and H, magenta). Nevertheless, aged animals showed a lower CTXc index for diacetyl even when the odorant concentrations were high (Fig. 1B, magenta). Similarly, the dispersion indices were high at high concentrations of isoamyl alcohol and benzaldehyde but low at high concentrations of diacetyl (Fig. 1D, G, and J, magenta). The time-course experiment of chemotaxis toward diacetyl showed that *E. coli*-fed animals experienced a marked reduction of CTXc and CTXi indices at D3 of adulthood. In contrast, the dispersion index remained relatively high at D3 (Supplementary Fig. 1). Interestingly, the timing of the chemotaxis decline was correlated with the end of self-reproduction (Supplementary Fig. 1). Collectively, we concluded that although aged animals have defects in the sensation of all three odorants, the severity of the sensory loss is odorant-dependent.

### Feeding with lactic acid bacteria maintains diacetyl chemotaxis in aged animals

To address the effects of diet on the age-dependent chemotaxis decline, we used *Lactobacillus reuteri* (*L. reuteri*) as an alternative diet because we have previously shown that switching animals’ diet from *E. coli* to *L. reuteri* during adulthood prevents age-dependent thermotaxis decline^12^. *L. reuteri* does not support the development of *C. elegans* (Supplementary Fig. 2). To avoid the dietary effects on the development, animals were fed with *E. coli* until D1 and then with *E. coli* or *L. reuteri* until day 5 of adulthood (D5). Compared to *E. coli*-fed aged animals, *L. reuteri*-fed aged animals showed better sensation (CTXi index) for diacetyl (Fig. 1C, light blue) and isoamyl alcohol (Fig. 1F, light blue) but not for benzaldehyde (Fig. 1I, light blue). Moreover, *L. reuteri*-fed aged animals maintained a higher CTXc index toward diacetyl. These results suggest that similar to what we previously saw in the age-dependent loss of odorant sensing, maintenance of chemotaxis behavior through diet acts in an odorant-specific manner.

**Figure 2:**
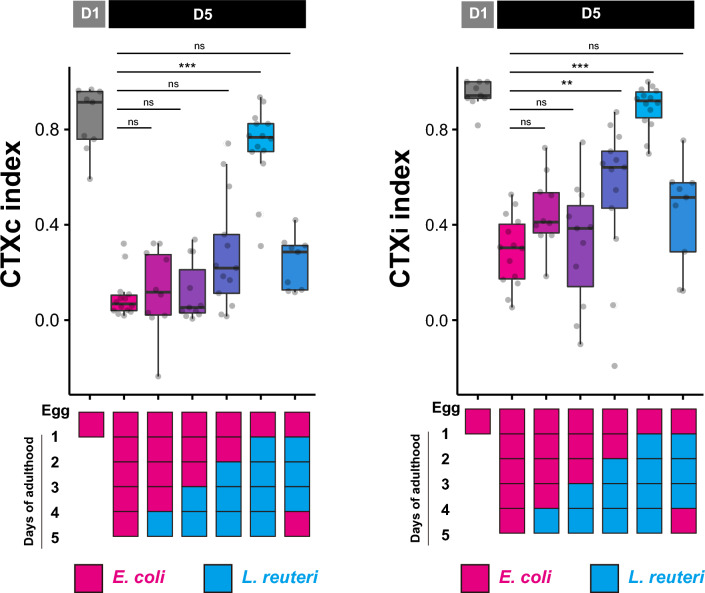
Chemotaxis of aged animals fed with *E. coli* or *L. reuteri* for different duration. Chemotaxis indices of animals fed different bacterial diets toward 0.1% diacetyl. All animals were fed *E. coli* until D1. D5 animals were fed with *E. coli* (magenta) or *L. reuteri* (blue) as indicated. Statistical significance was determined by the Kruskal–Wallis and Steel post-hoc tests. ns, *p* > 0.05; **p < 0.01; ***p < 0.001.

We sought to determine that *L. reuteri*-induced maintenance of chemotaxis in aged animals is due to long-term dietary effects and not to short-term olfactory adaptation, which occurs at a timescale of hours^16^. Because both age and diet dramatically affected the chemotaxis behavior toward diacetyl, we used a 0.1% concentration of diacetyl in all subsequent experiments. We sequentially switched the animals’ diet from *E. coli* to *L. reuteri* at different timings and found that a minimum of four consecutive days of feeding with *L. reuteri* was required to fully maintain high CTXc and CTXi indices of D5 animals (Fig. 2). On the other hand, switching diets from *L. reuteri* to *E. coli* one day prior to the assay was sufficient to decrease both CTXc and CTXi indices. To rule out the possibility that the age-dependent chemotaxis decline is due to olfactory adaptation, we tested a mutant strain of *egl-4*, which encodes a GMP-dependent protein kinase required for olfactory adaptation of benzaldehyde chemotaxis^16^. Since *egl-4* mutants displayed lower chemotaxis ability toward 0.1% diacetyl and benzaldehyde even at D1 (Supplementary Fig. 3), we used 1% of odorant instead. At this concentration, *egl-4* mutants showed age-dependent chemotaxis decline (Supplementary Fig. 3). These results imply that age- and dietary modulation of chemotaxis is unlikely attributed to olfactory adaptation.

### Bacterial diets modulate the expression of *odr-10* receptors via its promoter

**Figure 3:**
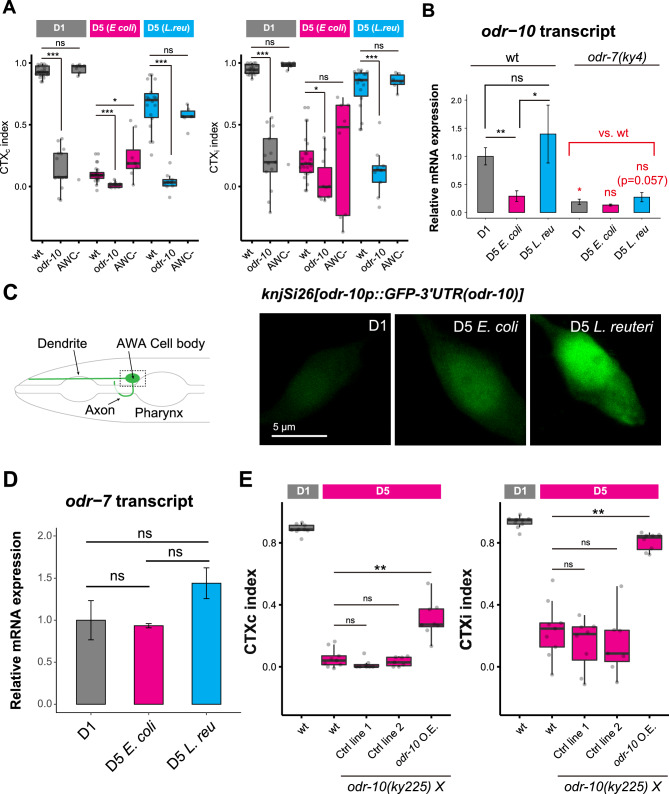
Diet modulates the *odr-10* expression of aged animals. (**A**) Chemotaxis indices of wild-type, *odr-10* mutants (CX3410), and AWC-ablated animals (IK2808) toward 0.1% diacetyl. All animals were fed *E. coli* until D1. D5 animals were fed with *E. coli* or *L. reuteri* from D1. (**B** and **D**) Relative mRNA expressions of *odr-10* in the wild-type and *odr-7* mutants (CX4) (**B**) and *odr-7* in the wild type (**D**). Aged animals were fed *E. coli* or *L. reuteri* from D1 to D5. For each condition, three biological replicates were tested. *cdc-42* was used as an internal reference. (**C**) Imaging of the GFP fluorescence in the AWA cell body of D1, *E. coli*-fed D5, and *L. reuteri*-fed D5 animals carrying the *odr-10*p::GFP::3’UTR (*odr-10*) single-copy transgene (NUJ393 *knjSi26*). Schematic diagram of the AWA neuron and representative fluorescent images are shown. The data are quantified in Supplementary Fig. 4. (**E**) Chemotaxis indices of D1 and *E. coli*-fed aged animals overexpressing *odr-7p::odr-10* (*odr-10* O.E.) in the *odr-10(ky225)* background. Both controls (Ctrl) and overexpression lines express hygromycin-resistant genes for selecting transgenic animals. In all panels, the statistical difference was determined using the Kruskal–Wallis and Steel post-hoc tests. ns, *p* > 0.05; *p < 0.05; **p < 0.01; ***p < 0.001.

We asked whether *L. reuteri*-fed aged animals can achieve chemotaxis through the same molecular and cellular mechanisms as young animals. In young animals, low concentrations (0.1%) of diacetyl are sensed by the odorant receptor ODR-10, specifically expressed in the AWA olfactory neurons^10^. At a higher concentration of diacetyl (10%), both AWA and AWC olfactory neurons redundantly regulate chemotaxis toward diacetyl^5^ (Colbert and Bargmann 1995). To determine whether AWA via *odr-10* or AWC neurons mediate the chemotaxis in the *L. reuteri*-fed aged animals, we compared diacetyl chemotaxis in *odr-10* mutants and AWC-ablated animals. AWC was ablated by overexpressing a split caspase under the control of an AWC-specific promoter^17^. Consistent with previous results^10^, chemotaxis of young animals toward diacetyl required *odr-10* and was independent of AWC neurons (Fig. 3A, D1). Similar to that of D1 animals, the chemotaxis of *L. reuteri-fed* aged animals was dependent on *odr-10* and independent of AWC neurons (Fig. 3A, D5). These results indicate that *L. reuteri* feeding maintains diacetyl chemotaxis through the odorant receptors in the AWA neurons instead of alternative recruitment of the AWC neurons.

Because *odr-10* was required for diacetyl chemotaxis in *L. reuteri*-fed aged animals, we asked whether dietary changes during aging influenced *odr-10* gene expression. The expression of the *odr-10* transcripts decreased during aging when animals were fed *E. coli*, while its levels were maintained in *L. reuteri*-fed aged animals (Fig. 3B, wt). To determine whether *odr-10* expression is regulated by its promoter, we measured the fluorescence of a single-copy *odr-10* transcriptional reporter (*knjSi26[odr-10p::GFP::3’UTR (odr-10)]*) in the AWA cell body. The fluorescence was higher in *L. reuteri*-fed aged animals than in *E. coli*-fed aged animals (Figs. 3C and Supplementary Fig. 4). Another multi-copy transcriptional reporter (*kyIs37[odr-10p::GFP::3’UTR (unc-54)]*) with a different 3’UTR showed the same trend of increased GFP signal in *L. reuteri*-fed aged animals (Supplementary Fig. 4), suggesting that diet affects *odr-10* expression through its promoter. We note that the GFP fluorescence was increased from D1 to D5 in *E. coli*-fed animals in both *knjSi26* and *kyIs37* (Fig. 3C and Supplementary Fig. 4) transgenes, unlike the trend we observed in the endogenous *odr-10* transcripts (Fig. 3B). The apparent increase in GFP fluorescence in aged animals is likely due to the accumulation of stable free GFP proteins during aging because the GFP transcripts in *knjSi26[odr-10p::GFP::3’UTR (odr-10)]* showed no alteration during aging (Supplementary Fig. 4) and GFP fused to ODR-10 decreased from D1 to D5 (see Supplementary Fig. 9). Given that the endogenous *odr-10* transcript, but not the GFP transcript under the control of *odr-10* promoter and 3’UTR, decreased during aging, the *odr-10* transcript might undergo post-transcriptional regulation. While aging and bacterial diet affect the expression of *odr-10* transcripts, they appear to do so at distinct stages: post-transcriptional and promoter activity regulation.

What lies upstream of *odr-10* expression under the aged, *L. reuteri*-fed conditions? In young animals, *odr-10* expression is regulated by the nuclear hormone receptor, ODR-7^10,18^. The expression of *odr-10* transcripts in both D1 and D5 animals was dependent on *odr-7* irrespective of diet (Fig. 3B). However, *odr-7* transcripts in aged *L. reuteri*-fed animals did not differ from that of *E. coli*-fed aged animals (Fig. 3D), suggesting that the ODR-7 activity instead of transcription might be regulated.

We next asked whether increasing the *odr-10* expression is sufficient to confer high chemotaxis ability in aged animals. Thus, we generated a strain overexpressing *odr-10* under the control of the *odr-7* promoter and tested its chemotaxis in aged animals. The *odr-10* overexpression restored the CTXi index of *E. coli*-fed aged animals, but the CTXc index was not comparable to young animals (Fig. 3E). On the other hand, the *odr-10* overexpression did not affect the chemotaxis of young animals (Supplementary Fig. 5). These results suggest that increasing *odr-10* expression is sufficient to restore an aged animal’s sensitivity toward diacetyl but insufficient to restore its ability to complete chemotaxis behavior.

### Food deprivation maintains high chemotaxis ability in aged animals

**Figure 4:**
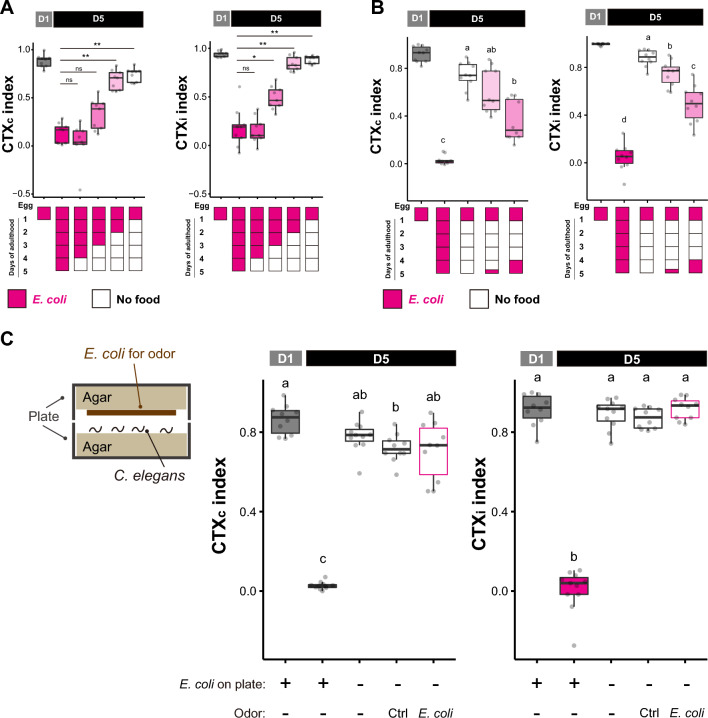
Food deprivation ameliorates chemotaxis decline. (**A**, **B**) Chemotaxis indices of animals with different feeding conditions toward 0.1% diacetyl. All the animals were fed with *E. coli* until D1. (**A**) Different durations of food deprivation. Dietary conditions are indicated in the schematic. Statistical significance was determined by the Kruskal–Wallis and Steel post-hoc tests. ns, *p* > 0.05; *p < 0.05; **p < 0.01. (**B**) The effect of re-feeding. Food-deprived animals were fed with *E. coli* 6 h or 24 h prior to the chemotaxis assays. (**C**) The effect of *E. coli* odor. The schematic shows the experimental setup to expose animals to the buffer (Ctrl) or *E. coli* odor. (**B** and **C**) Statistical significance was determined by comparing all pairs with the Kruskal–Wallis and Steel Dwass post-hoc tests. Different alphabets indicate significant statistical differences.

*C. elegans* is shown to ingest both *E. coli* and *L. reuteri*^19^. We sought to understand whether the maintenance of chemotaxis ability in *L. reuteri*-fed aged animals is due to the presence of a beneficial effect provided by *L. reuteri* or the absence of a detrimental effect derived from *E. coli*. To distinguish these possibilities, we assessed whether aged animals under food deprivation would mimic either behavioral phenotypes presented by *L. reuteri*-fed or *E. coli*-fed conditions. We found that aged animals under food deprivation maintained a higher chemotaxis ability toward diacetyl than animals fed with *E. coli* (Fig. 4A, magenta vs. white box plots), suggesting that feeding *E. coli* decreases the animals’ chemotaxis ability during aging. When tested for chemotaxis toward isoamyl alcohol and benzaldehyde, aged animals under food deprivation did not show recovery in chemotaxis ability (Supplementary Fig. 6). We further characterized the time-course relationship between food deprivation and age-dependent chemotaxis decline. Similar to *L. reuteri*-fed condition, food deprivation for at least three days was required to observe high chemotaxis ability at D5 (Fig. 4A). We also asked whether re-feeding food-deprived animals with *E. coli* could, in turn, reduce their chemotaxis performance, and examined re-feeding 6 or 24 h prior to the assay at D5. Although 6 h of re-feeding slightly reduced the CTXi index, further reduction of CTXi and CTXc indices were observed by 24 h of re-feeding (Fig. 4B). The time scale of the effect and the requirement of food consumption agrees with the former statement that the modulation of chemotaxis behavior is not due to chemosensory adaptation. Consistent with this notion, exposure to the *E. coli* odor did not decrease the chemotaxis ability of aged animals cultured without food (Fig. 4C).

We next asked whether food deprivation maintains chemotaxis in aged animals through caloric restriction. To assess this possibility, we tested the mutants of *eat-2*, which encodes an acetylcholine receptor expressed in pharyngeal muscles whose defects cause caloric restriction by reducing the animal’s feeding efficiency^20^. We found that *eat-2* mutants did not show high chemotaxis ability at D5 (Supplementary Fig. 7), suggesting that the effect of food deprivation tested under our conditions is distinct from the effect of caloric restriction.

Because dietary deprivation is known to extend lifespan^21^, we measured the lifespan of food-deprived and *E. coli*-fed animals. We found that food*-*deprived animals had a 30.6% longer lifespan than *E. coli*-fed animals (Supplementary Fig. 8, Supplementary Table 2). This increase in lifespan does not sufficiently explain the higher chemotaxis ability of food-deprived aged animals because *E. coli*-fed D3 animals already displayed significantly less chemotaxis ability (Supplementary Fig. 1) compared to food-deprived D5 animals. Thus, the high chemotaxis performance of food-deprived aged animals does not appear to be a secondary effect of a longer lifespan.

### *daf-16* is required for the chemotaxis of aged animals under food deprivation

Like *L. reuteri*-fed conditions, food deprivation increased the endogenous *odr-10* transcripts and *odr-10*p::GFP reporter expression in the wild-type animals (Fig. 6A–C, wt), suggesting that *L. reuteri* and food deprivation might maintain the chemotaxis behavior through the same mechanism. Furthermore, ODR-10 protein fused to GFP also showed higher expression in food-deprived aged animals than in *E. coli*-fed aged animals (Supplementary Fig. 9), which correlated to the *odr-10* transcript expression and chemotaxis behavior. *odr-7* was required for the chemotaxis behavior of food-deprived aged animals (Supplementary Fig. 10), implying that *odr-7* regulates the chemotaxis through *odr-10* expression like young animals.

**Figure 5:**
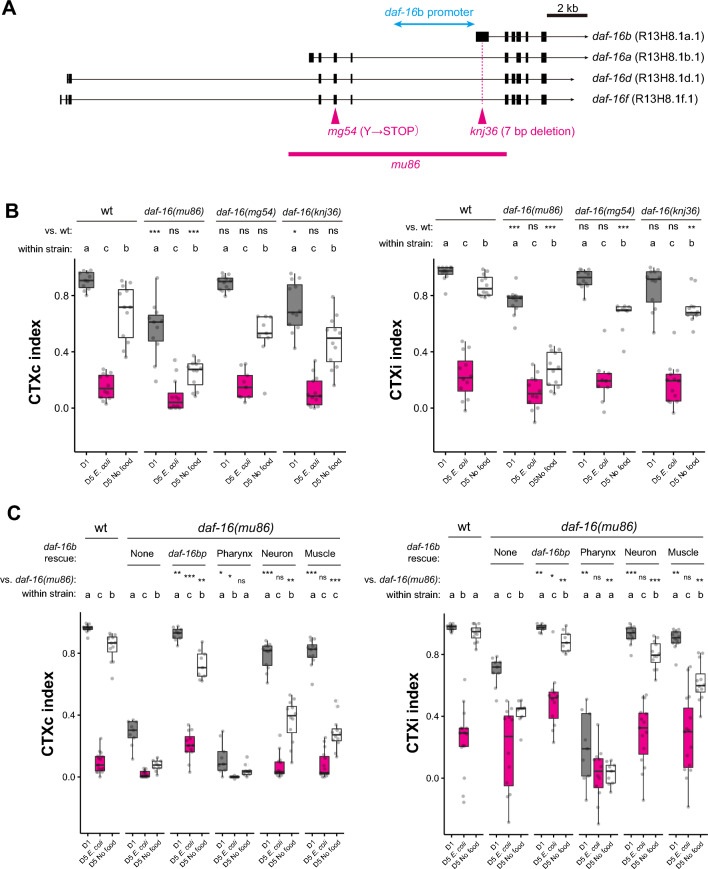
*daf-16* is required to maintain the chemotaxis ability of aged food-deprived animals (**A**) Schematic representation of *daf-16* isoforms and *daf-16 a*lleles. The black box, exon; black line, intron; magenta lines, deleted region; arrowheads, point mutations; light blue arrow, the promoter used for the rescue experiment. (**B** and **C**) Chemotaxis indices of D1, *E. coli*-fed D5, and food-deprived D5 animals with indicated genotypes toward 0.1% diacetyl. (**B**) The effect of different alleles of *daf-16* on the chemotaxis. (**C**) Tissue-specific rescue of *daf-16*. *daf-16b* cDNA was expressed in the *daf-16(mu86)* background under the control of *daf-16b* promoter, *myo-2* promoter (pharynx), *rgef-1* promoter (neuron), or *myo-3* promoter (muscle). (**B** and **C**) Statistical differences in all the pairs were determined within the same genotype using the Kruskal–Wallis and Steel Dwass post-hoc tests. Different alphabets indicate significant statistical differences. The same conditions across the genotype were compared to the wild type using the Kruskal–Wallis and Steel post-hoc tests. ns, *p* > 0.05; *p < 0.05; **p < 0.01; ***p < 0.001*.*

To further investigate which genes are responsible for the food deprivation-dependent high chemotaxis ability in aged animals. We focused on the FOXO transcription factor DAF-16 because we have previously shown that DAF-16 is involved in the dietary effect on thermotaxis behavior in *C. elegans*^12^. The young D1 *daf-16(mu86*) null mutants showed marginal defects in chemotaxis (Fig. 5A and B). On the other hand, the high chemotaxis ability of food-deprived aged animals was completely dependent on *daf-16*, as *daf-16(mu86*) mutants performed poorly in both CTXc and CTXi indices (Fig. 5B). Since *daf-2* encoding insulin/IGF-1 receptor negatively regulates DAF-16^13^, we tested whether *daf-2* mutants phenocopied food deprivation. Indeed, *daf-2* aged animals showed higher chemotaxis ability than the wild-type counterparts, even under *E. coli*-fed conditions (Supplementary Fig. 7).

We examined the tissue and isoform specificity of *daf-16*. The *daf-16* genomic locus encodes several different isoforms with different N-termini transcribed from unique promoters (Fig. 5A)^22–24^. These isoforms are expressed in different tissues: *daf-16a* and *daf-16f* are expressed in the hypodermis, muscle, neurons, and intestines, while *daf-16b* is expressed primarily in the neurons, pharynx, and muscles^22,24–27^. We tested lines carrying isoform-specific mutations of *daf-16*: 1) *daf-16(mg54)* introduced with a premature stop codon to *daf-16a, d, f*; 2) *daf-16(knj36)* introduced 7-bp deletion to *daf-16b* isoform (Fig. 5A). Neither *daf-16(mg54)* nor *daf-16(knj36)* mutants showed substantial defects in chemotaxis (Fig. 5B), suggesting that the existence of either isoform is sufficient to maintain high chemotaxis of aged animals under food deprivation. We further validated that the *daf-16b* isoform is sufficient to induce high chemotaxis ability by demonstrating that a transgenic line consisting of a genomic DNA of the *daf-16b* isoform, including its endogenous promoter (*knjSi17[daf-16bp::daf-16b]*) fully rescued the phenotype of *daf-16(mu86)* mutant (Fig. 5C, *daf-16bp*).

To determine in which tissue *daf-16* functions, we performed rescue experiments for the tissue where *daf-16b* is expressed. The expression of *daf-16* in muscles and neurons partially rescued the full-body knockout of *daf-16*, while the expression in the pharynx did not (Fig. 5C), suggesting that *daf-16* might be functioning in neurons and/or muscle to elicit chemotaxis under food deprivation.

**Figure 6:**
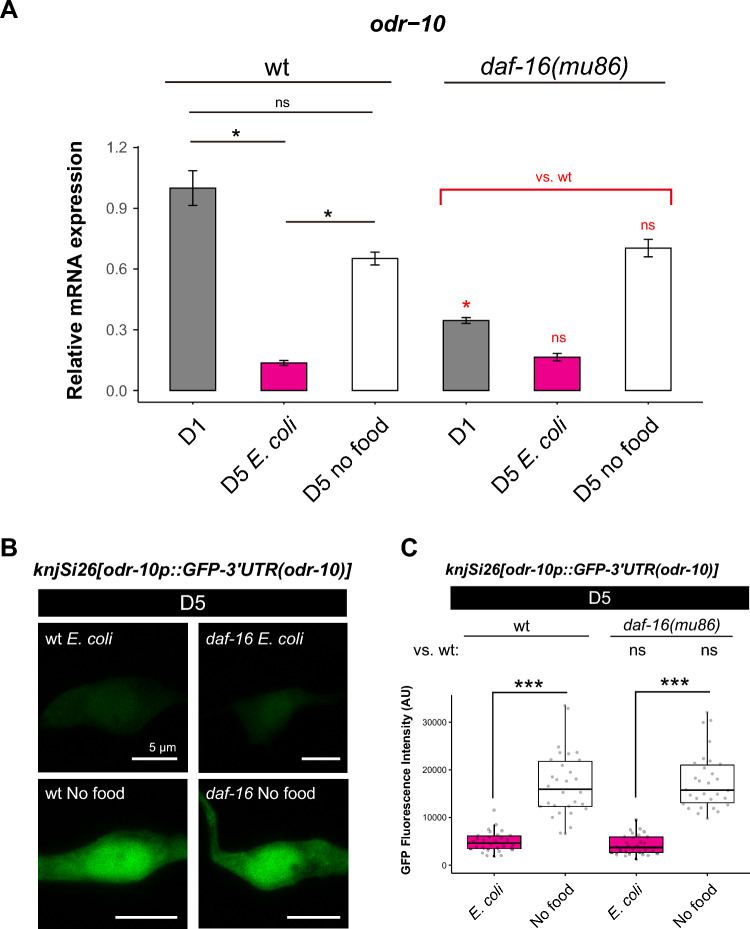
Food-deprived animals exhibit a higher level of *odr-10* expression compared to fed animals. (**A**) Relative expression of *odr-10* mRNA using quantitative PCR. (**B** and **C**) Representative images (**B**) and quantification (**C**) of the *odr-10* transcriptional reporter (*knjSi26[odr-10p::GFP::3’UTR (odr-10)]*) in *E. coli*-fed D5 and D5 food-deprived animals in the wild-type and *daf-16(mu86)* background. Animals were fed with *E. coli* until D1. For (**A**) and (**C**), statistical difference was determined using the Kruskal–Wallis and Steel Dwass post-hoc tests for the indicated pairs. ns, *p* > 0.05; *p < 0.05, ***p < 0.001*.* The scale bar in (**B**) indicates 5 µm.

As DAF-16 is a transcription factor that functions at least partially in neurons, we asked whether *daf-16* might regulate the expression of *odr-10.* The higher expression of *odr-10* in the food-deprived condition did not depend on *daf-16* (Fig. 6A–C, *daf-16*), suggesting that *daf-16* was acting downstream or independently of the *odr-10* expression to affect the chemotaxis behavior of food-deprived aged animals.

## Discussion

We established a model to examine the effects of bacterial feeding and food deprivation on the age-dependent decline in chemotaxis of *C. elegans*. Using dose–response curves of three different odorants, we systematically characterized the effect of aging and diet. The onset of age-dependent decline in odor sensation (CTXi) and chemotaxis completion (CTXc) differ, suggesting that distinct mechanisms underlie these phenomena. Consistent with this notion, overexpressing *odr-10* in aged animals fully restored the CTXi index but not the CTXc index towards diacetyl chemotaxis. Migration behavior toward an odorant may contribute to complete chemotaxis because the dispersion index reflecting the animals’ exploration behavior declined along with the CTXc index. The lack of migration behavior toward low concentrations of odorant is distinct from locomotion defects because aged animals maintained the ability to move toward high concentrations of odorants. The locomotion defects due to muscle deterioration are reported to emerge at a later stage^7^.

The effect of *L. reuteri* feeding and food deprivation required a few days to manifest, suggesting that dietary effects are not simply due to canonical forms of sensory adaptations, which occur within a few hours^28^. Furthermore, although adaptation of the chemotaxis behavior toward diacetyl is regulated by AWC sensory neurons^28^, AWC ablation did not affect the chemotaxis behavior of *E. coli*-fed or *L. reuteri*-fed aged animals. Since larvae are exposed to *E. coli* odorants from the beginning of their lifetime, it is unlikely that the same form of adaptation happens only during aging to decrease chemotaxis ability. These results imply that age-dependent chemotaxis decline undergoes a different mechanism from sensory adaptation. Instead, our data suggests that ingestion of some factors in *E. coli* may affect the internal state of animals, which causes the reduction of *odr-10* expression and, consequently, behavioral decline. On the other hand, animals fed with *L. reuteri* or without food were not exposed to these factors and maintained the *odr-10* expression and chemotaxis ability. Internal nutritional state regulation may involve inter-tissue communication from the intestine to the neurons. For example, the intestinal expression of insulin-like peptides (ILPs) induces feeding-state-dependent regulation of chemoreceptor *srh-24* expression in the ADL neurons^29^. In starved young animals, the intestine-derived neuropeptide INS-1 targets DAF-16 in the AWC neurons and disrupts the thermotaxis behavior^30^.

While aging reduced chemotaxis ability towards all three odorants tested, diacetyl was affected the most. Moreover, dietary intervention by feeding *L. reuteri* and complete food deprivation specifically improves diacetyl chemotaxis in aged animals. This phenomenon might be attributed to the evolutionary importance of diacetyl chemotaxis towards an animal’s survival, and as such, the circuit remains flexible for satiety inputs even as the animal ages. Indeed, others have shown that gonadal maturation only affects diacetyl chemotaxis but not chemotaxis of other odorants^31^.

Age-dependent chemotaxis decline can be caused by various processes, such as the malfunction of signaling pathways in the sensory neurons, defective synaptic transmission, and locomotion defects. Our findings nailed down that aging and dietary changes can affect diacetyl chemotaxis through the expression of *odr-10* encoding the diacetyl receptor. Indeed, chemoreceptor expression is shown to be state-dependent; the expression of the AWA chemoreceptor *str-44,* for example, is controlled by sensory and metabolic components of food^32^. Furthermore, increased diacetyl-evoked responses in the AWA neurons are also shown to directly correlate to an upregulation of ODR-10 in both dauer larvae and postdauer adults^33^. *L. reuteri* feeding and food deprivation maintained high *odr-10* expression in aged animals, most likely via the *odr-10* promoter. *odr-10* promoter region is predicted to contain an ODR-7-binding site based on the in-silico analysis^34^. We speculate that binding of ODR-7 to the *odr-10* promoter, but not *odr-7* expression, regulates *odr-10* during aging. ODR-7 is a nuclear hormone receptor, whose ligands can be steroid hormones, and steroid hormones are implicated to affect aging by modulating the lifespan of *C. elegans*^35,36^. Thus, age-dependent alteration in steroid hormones may affect behavior through the expression of the diacetyl receptor, ODR-10, in the primary sensory neurons, AWA. Unlike ODR-10 for diacetyl, the receptors for benzaldehyde and isoamyl alcohol are not well characterized. Since all three tested odorants showed a decline in sensitivity (CTXi) when fed with *E. coli*, the downregulation of odorant receptor gene expression might be a general phenomenon when aged animals are fed with *E. coli*. However, the mechanism of the age-dependent decline of the chemotaxis behavior toward benzaldehyde is attributed to the secondary, instead of primary, sensory neurons^37^. Thus, the molecular mechanisms underlying the age-dependent chemotaxis decline might depend on the odorants.

We found that the transcription factor DAF-16 is necessary to maintain high chemotaxis ability in food-deprived aged animals. *E. coli* might trigger the activation of the insulin-signaling DAF-16 pathway during aging. A part of the forkhead DNA binding domain in DAF-16B is encoded by unique exons^24^. Because of this difference in the DNA binding domain, DAF-16B may regulate the expression of its own set of genes that differs from that of DAF-16A/D/F. Isoform-specific knockouts of either *daf-16a/d/f* or *daf-16b* did not have comparable phenotypes to null mutants. This result suggests that the existence of either isoform is sufficient. Thus, these different isoforms might share the target genes involved in the chemotaxis of aged animals. A nutritional state can directly regulate chemoreceptor expression via intermediaries such as DAF-7 in the TGFβ pathway in young animals^38^. The nutritional state also regulated the *odr-10* expression of aged animals. However, quantification of transcripts and transcriptional GFP reporter assays argued that *daf-16* did not regulate *odr-10* expression despite the existence of a putative binding site for DAF-16 809bp ahead of the *odr-10* start codon^39^. Instead, DAF-16 may regulate the transcriptional activity of molecules downstream of ODR-10 required for the chemotaxis in AWA. It is also possible that *daf-16* regulates the activity of the interneurons downstream of AWA, which are required to complete chemotaxis. Our experiments where restoration of *daf-16* expression in either neuronal or muscular tissues rescues the high chemotaxis of food-deprived animals point to such a possibility. Indeed, *daf-16* is also known to be required for the proper neurite extension of AIY interneurons, downstream of AWA during development^26^. Further studies are required to address the target of DAF-16 in regulating the chemotaxis of aged animals.

## Materials and methods

### *C. elegans* culture and strains

*C. elegans* strains were maintained on nematode growth medium (NGM) plates seeded with the *Escherichia coli* (*E. coli*) strain OP50^11^. The Bristol N2 strain was used as the wild type. Animals were maintained at 23 °C unless stated otherwise. Temperature-sensitive *daf-2(e1370)* animals were maintained at 15 °C until adulthood. Strains used in this study were obtained from Caenorhabditis Genetics Center (CGC) or generated in our laboratory using a standard crossing or injection (Supplementary Table 3). Single copy-inserted *knjSi26[odr-10p::gfp::3’UTR (odr-10)]* allele targeting *cxTi10882* site on chromosome IV was generated as previously described^40^ using microinjection of the following plasmids: pKEN990 (*odr-10p::gfp::3’UTR (odr-10)* + Hyg^R^), pCZGY2750 (*eft-3p::Cas9* + sgRNA for *cxTi10882* site) provided by Dr. Yishi Jin, and three co-injection markers, pGH8(*rab-3p::mCherry*), pCFJ90(*myo-2p::mCherry*), and pCFJ104(*myo-3p::mCherry*), gifts from Dr. Erik Jorgensen. Animals with the desired single copy insertion were selected based on the hygromycin resistance and the absence of co-injection markers. The transgenic strains for the *daf-16* rescue experiments were described previously^19^.

### Preparation of bacterial plates

*E. coli* OP50 obtained from CGC was inoculated into Super Broth (32 g/L Bacto Tryptone, 2 g/L Bacto Yeast extract, 0.5 g/L NaCl, 0.5 mM NaOH) and cultured overnight at 37 °C without shaking. The lactic acid bacterial strain, *L. reuteri,* was provided by Megmilk Snow Brand company. *L. reuteri* was inoculated from glycerol stocks and cultured in MRS broth (BD) overnight at 37 °C without shaking. After culturing, bacterial cells were pelleted by centrifugation at 7000 × *g* at 4 °C. Bacteria were washed twice with sterile 0.9% NaCl solution and adjusted to a final concentration of 100 mg/mL in NG buffer (51 mM NaCl, 1 mM CaCl_2_, 1 mM MgSO_4_, 25 mM K-PO_4_).

### Preparation of young and aged animals fed with different diets

To synchronize animals for chemotaxis assays, eggs were prepared by bleaching gravid hermaphrodites using 0.5 × household bleach in 0.5 M NaOH. Roughly 150 ~ 200 eggs were transferred onto NGM plates seeded with *E. coli* OP50 and cultivated at 23 °C. To prevent their progenies from hatching, 25 µM of 5′-fluorodeoxyuridine-2′-phosphate (FUdR) was applied to L4-stage animals 48 h after egg preparation. The plates were incubated at 23 °C for 24 more hours after FUdR treatment to obtain animals of day one of adulthood (D1). To prepare day 5 adult animals (D5), D1 animals were washed with NG buffer (51 mM NaCl, 1 mM CaCl_2_, 1 mM MgSO_4_, 25 mM K-PO_4_), transferred onto new *E. coli*-seeded, *L. reuteri*-seeded, or unseeded peptone-free NGM plates supplemented with 25 µM FUdR and incubated at 23 °C for four more days. Peptone-free plates were used to avoid the growth of residual bacteria. When necessary, animals were transferred to the indicated bacterial plates or unseeded plates during aging to switch diets. To test for the olfactory effects of *E. coli* in food-deprived conditions, animals were placed onto unseeded plates as described above, while the lids were replaced by *E. coli*-seeded plates or unseeded plates as a control.

### Population chemotaxis assay

Chemotaxis assays were performed based on procedures described by Bargmann et al.^5^. The assays were performed on 90-mm agar plates containing 2% BactoAgar, 5 mM K-PO_4_, 1 mM CaCl_2_, and 1 mM MgSO_4_. Animals from cultivation plates were collected into glass tubes with M9 buffer (20 mM KH_2_PO_4_, 20 mM Na_2_HPO_4_, 8 mM NaCl_2_, and 20 mM NH_4_Cl), washed with M9 buffer, followed by washing with chemotaxis buffer (5 mM K-PO_4_, 1 mM CaCl_2_, and 1 mM MgSO_4_) three more times. Four microliters of attractant (diacetyl, benzaldehyde, or isoamyl alcohol) diluted in 100% ethanol and 4 µL of control solvent (100% ethanol) were spotted onto the plate as indicated in Fig. 1A. To immobilize animals once they reached the respective points, 1 µL of NaN_3_ was spotted on the points of attractant and control. Approximately 100–150 animals were transferred onto three spots in the middle of the assay plate and allowed to move for an hour. To stop the assay, chloroform was applied to the lid of the plate. The number of animals was counted to obtain the complete chemotaxis index (CTXc), incomplete chemotaxis index (CTXi), and dispersion index, as indicated in Fig. 1A. In the box plots, each data point represents the chemotaxis index obtained from a single chemotaxis plate.

For the dose–response curves, fitting was created using R following a 4-parameter logistic (4PL) model estimate^41^. Curves were generated only when the data was fit. Data with significantly lower values than those at the highest lower concentration on the Student’s t-test were excluded from the analysis to allow better curve fitting because animals were repelled from high concentrations of attractant odorant.

### Quantitative RT-PCR

Young animals were prepared similarly to the chemotaxis assay, but non-gravid young adult animals at 56 h post egg preparation were used to exclude the RNA of embryos. D5 animals were prepared as the chemotaxis assays. Before RNA extraction, animals were washed twice with M9 buffer for 20 min to remove bacteria on the surface and in the intestine and then stored overnight at − 80 °C. RNA was extracted from animals by applying RNAiso Plus reagent (Takara) and purified according to the manufacturer’s instructions.

RNA samples were diluted to 0.5 µg/10 µL in RNAase-free water and then reverse-transcribed into cDNA using the ReverTra Ace qPCR RT Master Mix with gDNA Remover (TOYOBO). Diluted cDNA was mixed with SYBR qPCR mix (TOYOBO) and gene-specific primers listed in Supplementary Table 4. Quantitative PCR reactions were run in the LightCycler® 96 Instrument (Roche) with the following program: Preincubation (95 °C for 60 s) × 1, (95 °C for 10 s, 60 °C for 30 s) × 40, (95 °C for 10 s, 65 °C for 60 s, 97 °C continuous) × 1. The relative expressions were determined using *cdc-42* as a reference gene, whose expression is stable during aging^42^, and analyzed using the LightCycler® 96 SW 1.1. software (Roche). Three technical replicates were analyzed for each biological replicate, and the average value was used. Three biological replicates were analyzed for each condition.

### Lifespan assay

Animals were synchronized by bleaching, as described above. After reaching the D1 stage, animals were transferred onto the peptone-free NGM plates with *E. coli* or no food. Each trial consists of ~ 100 animals, with ~ 50 animals per plate for the *E. coli*-fed condition and ~ 100 animals per plate for the food-deprived condition. The trials were repeated thrice (~ 300 animals per condition). Animals were transferred every other day using a sterilized platinum wire pick onto peptone-free, FUdR-supplemented plates. Animals were considered dead if they did not respond to gentle touch with the pick and considered censored when missing, having crawled off, having burrowed, or carrying internally hatched progeny. Results were analyzed using the OASIS2 software^43^ to obtain the Kaplan–Meier survival curve and statistical comparison between the groups.

### Confocal microscopy

Live animals with *odr-10p*::GFP transgenes were immobilized on a 1% agarose patch with 0.01 mM levamisole and observed under a microscope (Axio Examiner Z1; Zeiss) equipped with a 63x, Plan Apochromatic Oil objective lens (Zeiss), a confocal unit (LSM880-SU, Zeiss), a lighting device with Ar458,488,514/DPSS561/HeNe633 nm (HXP 120 V, Zeiss), and a camera (LSM BiG2, Zeiss). Image acquisition was controlled by the Zeiss ZEN software. The fluorescence images were captured with a 488 nm laser. Using FIJI^44^, the mean fluorescent intensity values of AWA soma were obtained.

### Statistical analysis

Graphs were generated using RStudio. In box plots, a box indicates the first and third quartiles with a median, and the whiskers are extended to the maximum and minimum values except for outliers outside the range of 1.5 times of the interquartile value. Statistical analyses were performed in RStudio, and statistical differences between groups were determined using Kruskal–Wallis as a non-parametric test. As post-hoc tests, the Steel test was used for all pairwise comparisons within a genotype, and Steel–Dwass was used for comparison against the control within a condition. Bar graphs are expressed as mean ± S.E.M. ns, *, **, and *** indicate *P-*values that are > 0.05, < 0.05, < 0.01, and < 0.001, respectively.

### Supplementary Information

Supplementary Figures.

Supplementary Tables.

## Data Availability

All data analyzed for this article and supplementary information are made available in this published article. Source data are provided in this paper.
