# Supplementary material for: Dietary E. coli promotes age-dependent chemotaxis decline in C. elegans
Source: Sci Rep. 2024 Mar 6;14:5529. doi: 10.1038/s41598-024-52272-4 (PMC10918063; doi:10.1038/s41598-024-52272-4)
Supplement: Supplementary file 1 — Supplementary Figures. [file 41598_2024_52272_MOESM1_ESM.pdf]

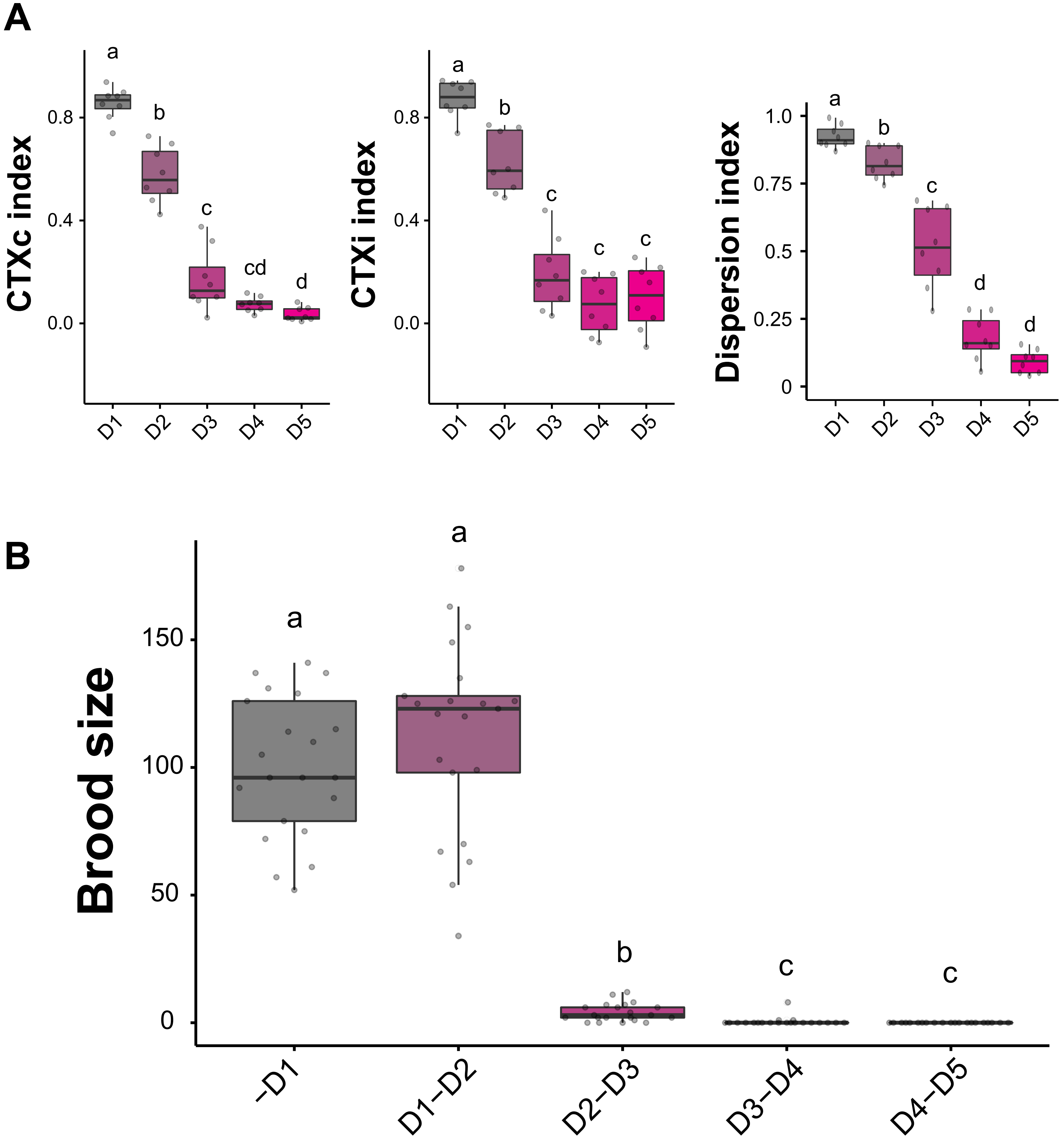

Supplementary Figure 1. Chemotaxis ability declines with age

(A) Chemotaxis indices and dispersion index of the *E. coli*-fed wild-type animals toward 0.1% diacetyl at different ages. **(B)** Brood size at different ages. L4 animals were singly transferred to a plate, cultured for 24 hours at 23°C, and the number of progeny was counted (-D1). Animals were transferred every 24 hours to count the brood size for each age. **(A and B)** Statistical significance was determined by comparing all pairs with the Kruskal–Wallis and Steel Dwass post-hoc tests. Different alphabets indicate significant statistical differences.

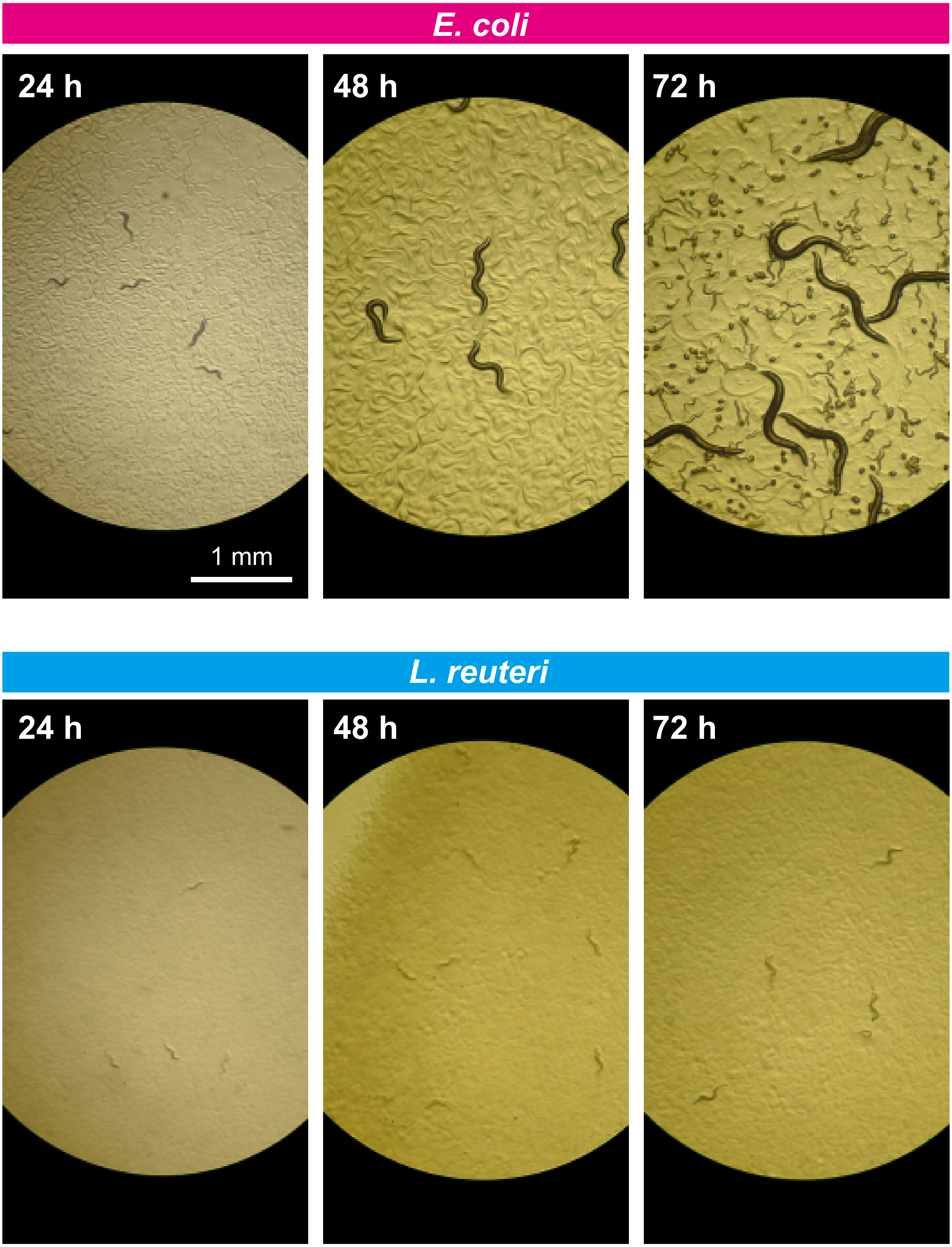

Supplementary Figure 2. ***L. reuteri*** **does not fully support the growth of animals**
Representative images of animals fed with *E. coli* or *L. reuteri* from eggs. Animals were incubated at 23°C after egg preparation by bleaching. Scale bar = 1 mm.

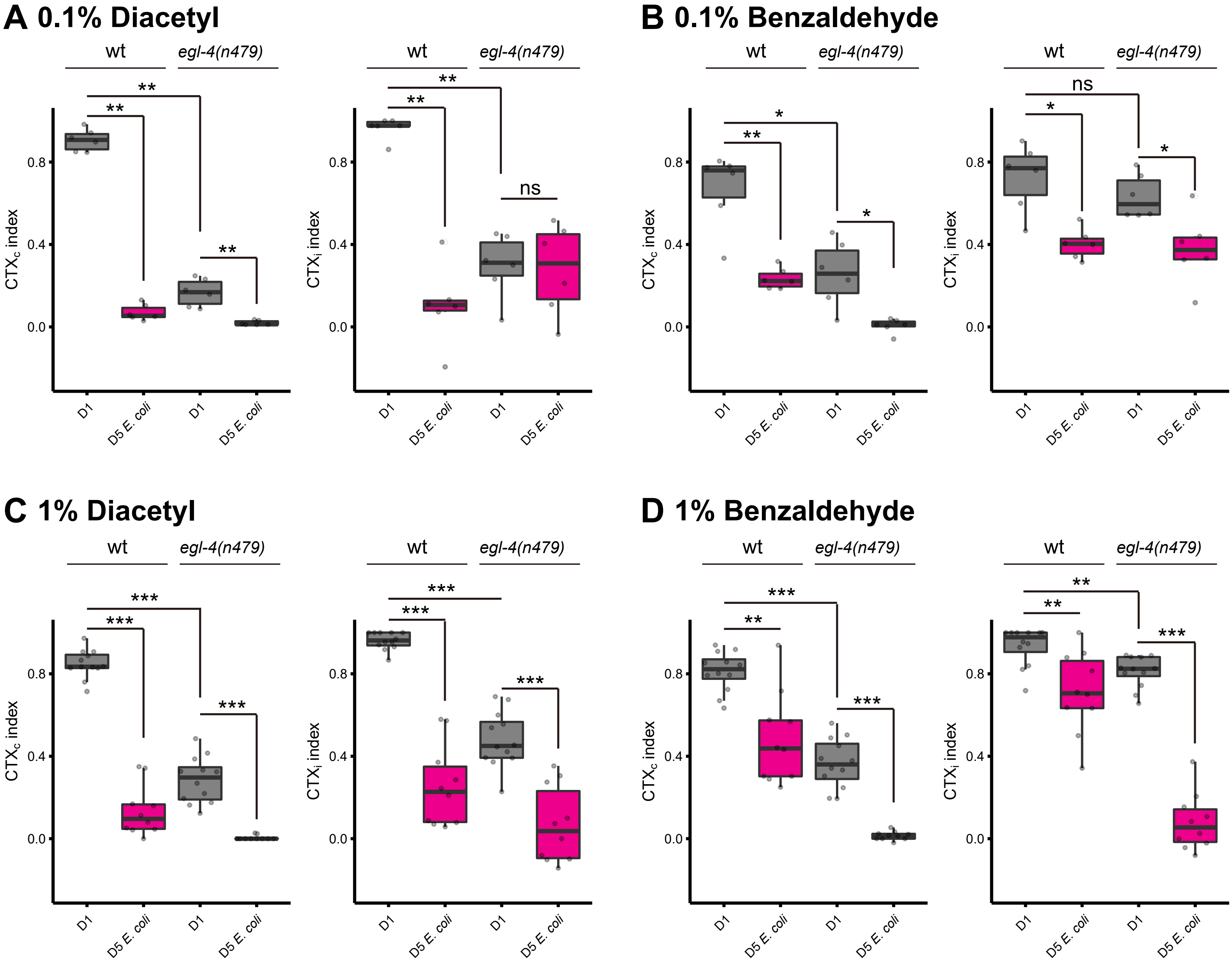

Supplementary Figure 3. *egl-4* mutants chemotaxis decline
Chemotaxis indices of D1 and D5 animals of the wild type and IK0571 *egl-4(n479)* with 0.1% diacetyl **(A)**, 0.1% benzaldehyde **(B)**, 1% diacetyl **(C)**, and 1% benzaldehyde **(D)**. Statistical significance was determined by the Kruskal–Wallis and Steel post-hoc tests. ns, $p>0.05$; *$p<0.05$; **$p<0.01$; ***$p<0.001$.

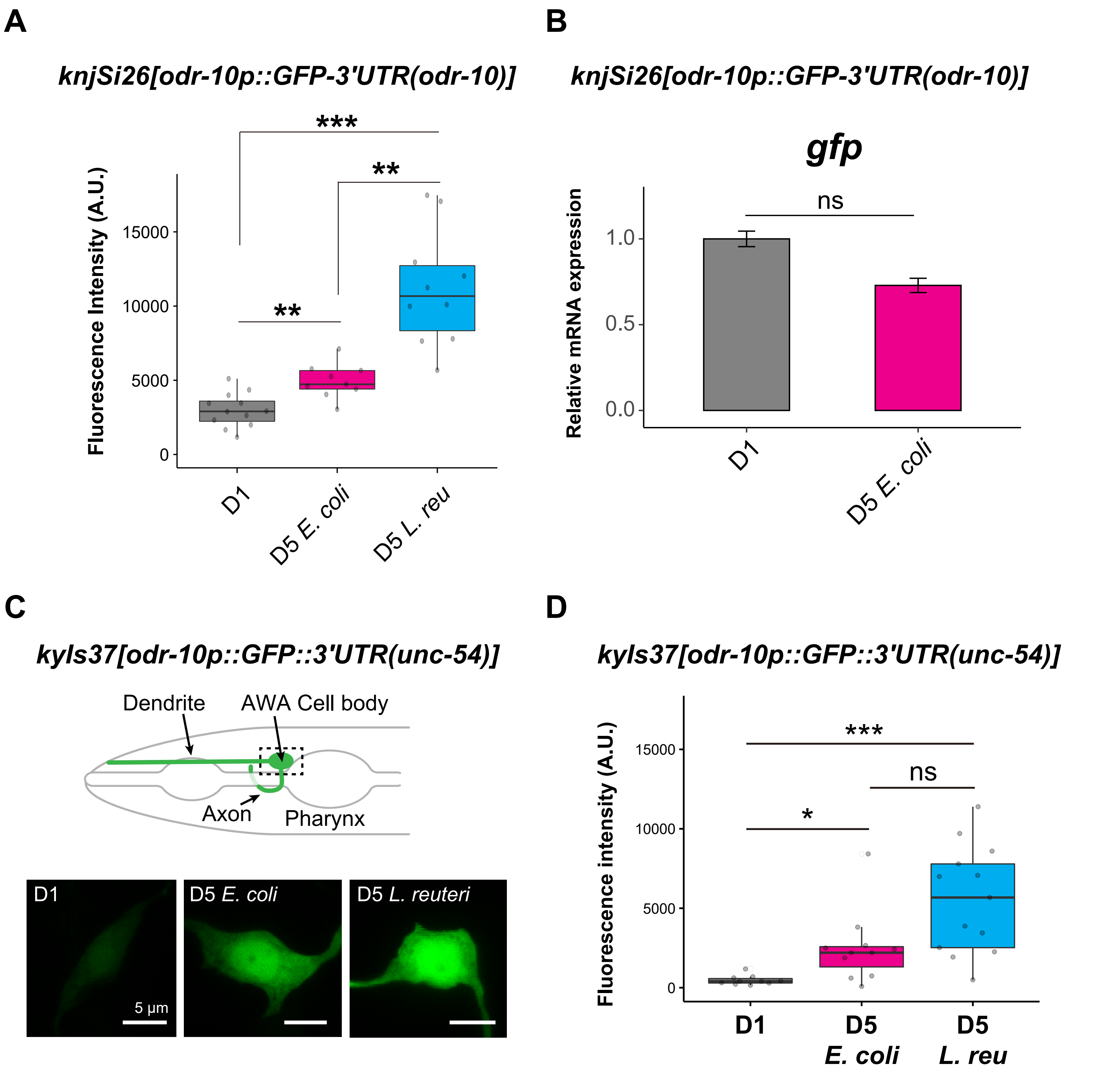

Supplementary Figure 4. Diet modulates the intensity of the *odr-10* transcriptional reporter of aged animals
(A and D) Quantitative analysis of the fluorescent intensity of the AWA cell body expressing the single-copy transcriptional reporter *knjSi26[odr-10p::GFP-3'UTR(odr-10)]* transgene (A) or the multi-copy transcriptional reporter *kyIs37[odr-10p::GFP::3' UTR (unc-54)]* transgene (D). Representative images of *knjSi26* strains are shown in Figure 3C. Aged animals were fed *E. coli* or *L. reuteri* from D1 to D5. D1, *E. coli*-fed D5, and *L. reuteri*-fed D5 animals were compared. Each data point represents the fluorescence intensity in the AWA cell body of a single animal. Statistical significance was determined by the Kruskal–Wallis and Steel post-hoc tests. ns, $p>0.05$; *$p<0.05$; **$p<0.01$; ***$p<0.001$. **(B)** Relative gfp mRNA expressions of D1 and *E. coli*-fed D5 animals. For each condition, three biological replicates were tested. *cdc-42* was used as an internal reference. Statistical significance was determined by the Mann-Whitney U test. ns, $p>0.05$. **(C)** Schematic diagram and representative confocal images of AWA cell bodies expressing the multi-copy transcriptional reporter *kyIs37[odr-10p::GFP::3' UTR (unc-54)]* transgene. Scale bar = 5 μm.

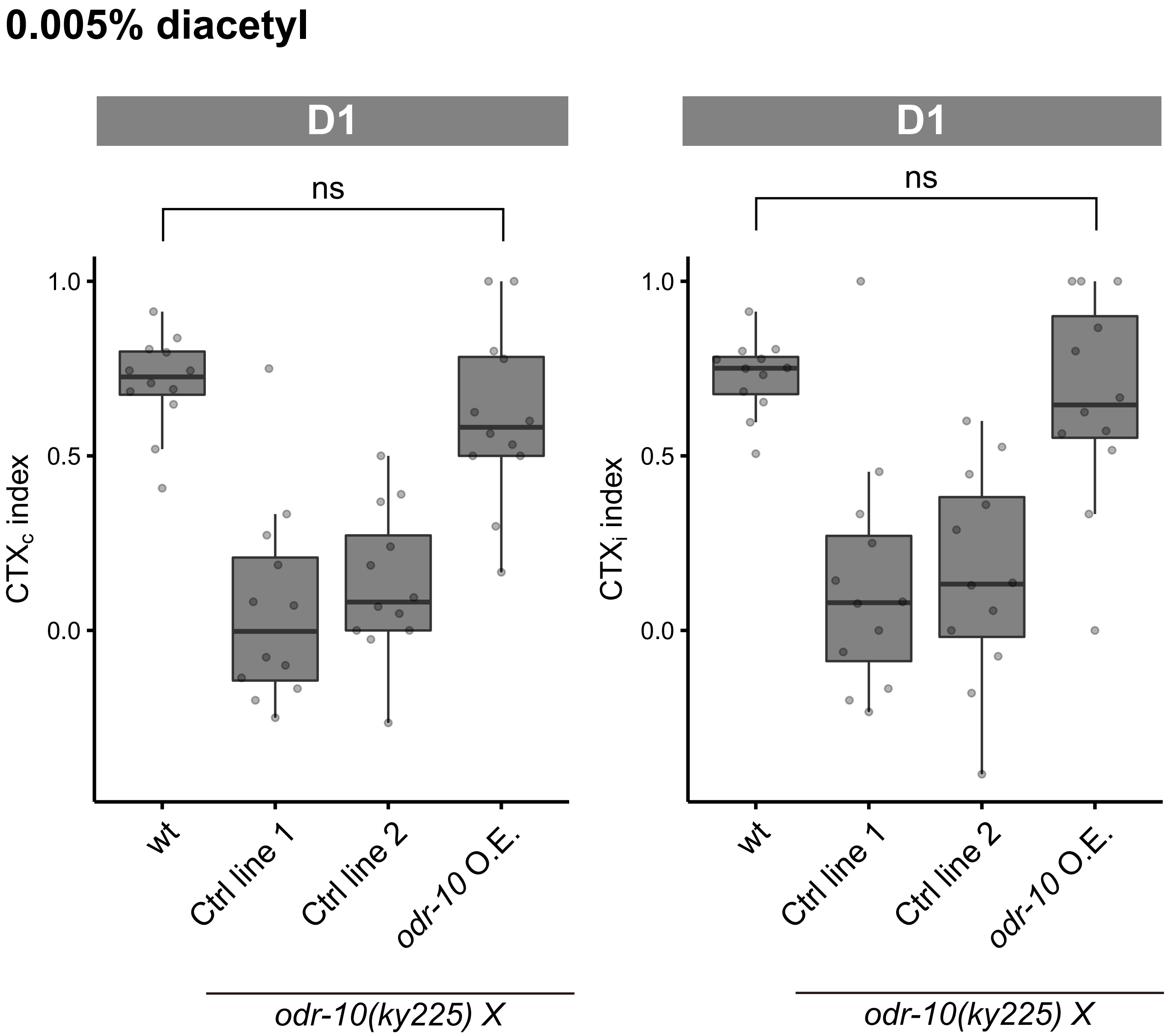

Supplementary Figure 5. *odr-10* overexpression does not enhance chemotaxis of D1 animals
Chemotaxis indices toward 0.005% diacetyl. *odr-7p::odr-10* was overexpressed in D1 animals in the *odr-10(ky225)* background (*odr-10* O.E.) . Both controls (Ctrl) and overexpression lines express hygromycin-resistant genes for selecting transgenic animals. The statistical difference compared to the wild type was determined using the Kruskal–Wallis and Steel post-hoc tests. ns, $p>0.05$.

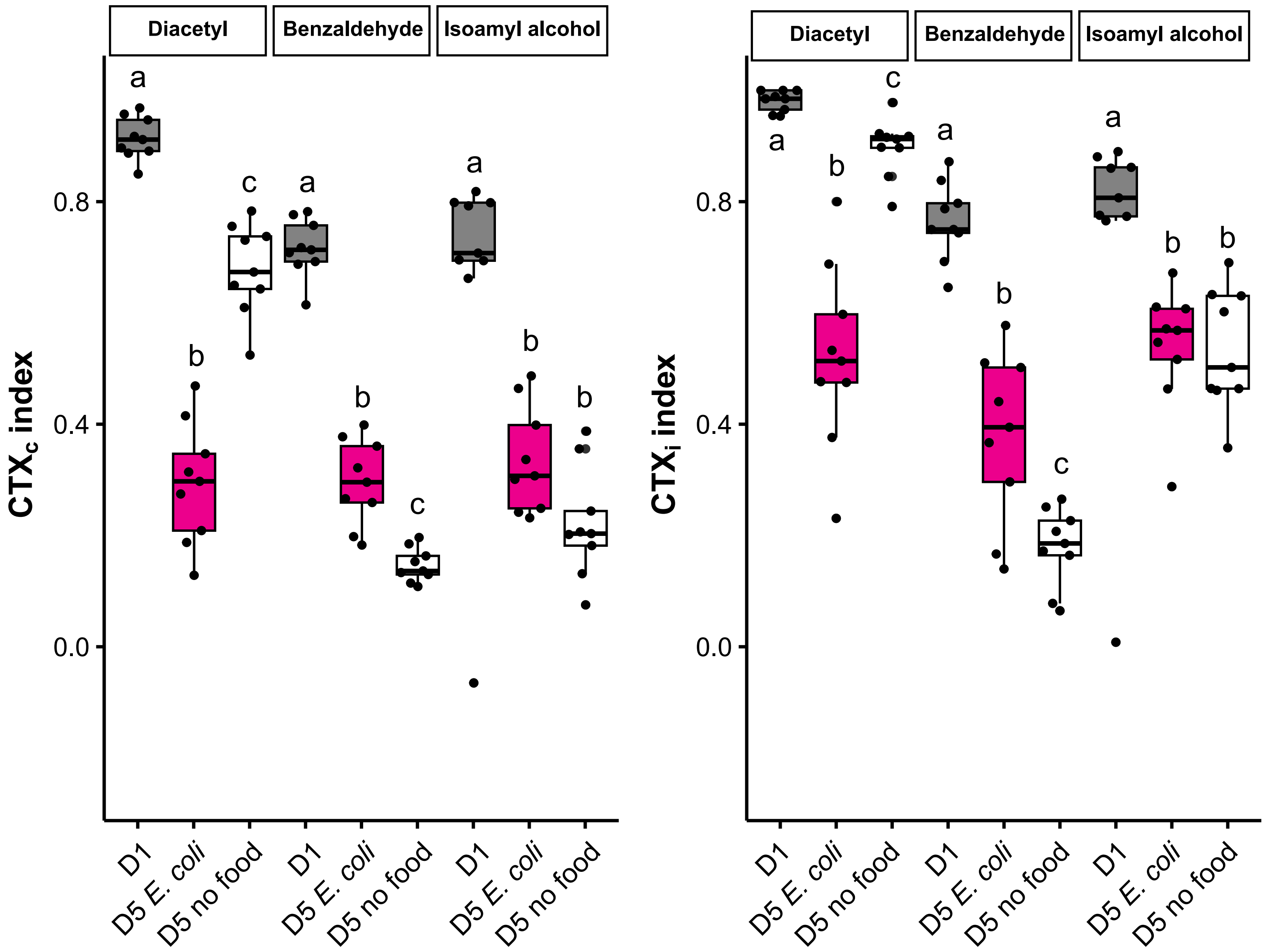

Supplementary Figure 6. The effect of food deprivation on the chemotaxis of aged animals toward different odorants

Chemotaxis indices of D1, D5 *E. coli*-fed, and D5 food-deprived animals with 0.1% diacetyl **(A)**, 0.1% benzaldehyde **(B)**, and 0.1% isoamyl alcohol **(C)**. Statistical significance was determined by all pairs in each odorant condition by the Kruskal-Wallis and Steel Dwass post-hoc tests. Different alphabets indicate significant statistical differences.

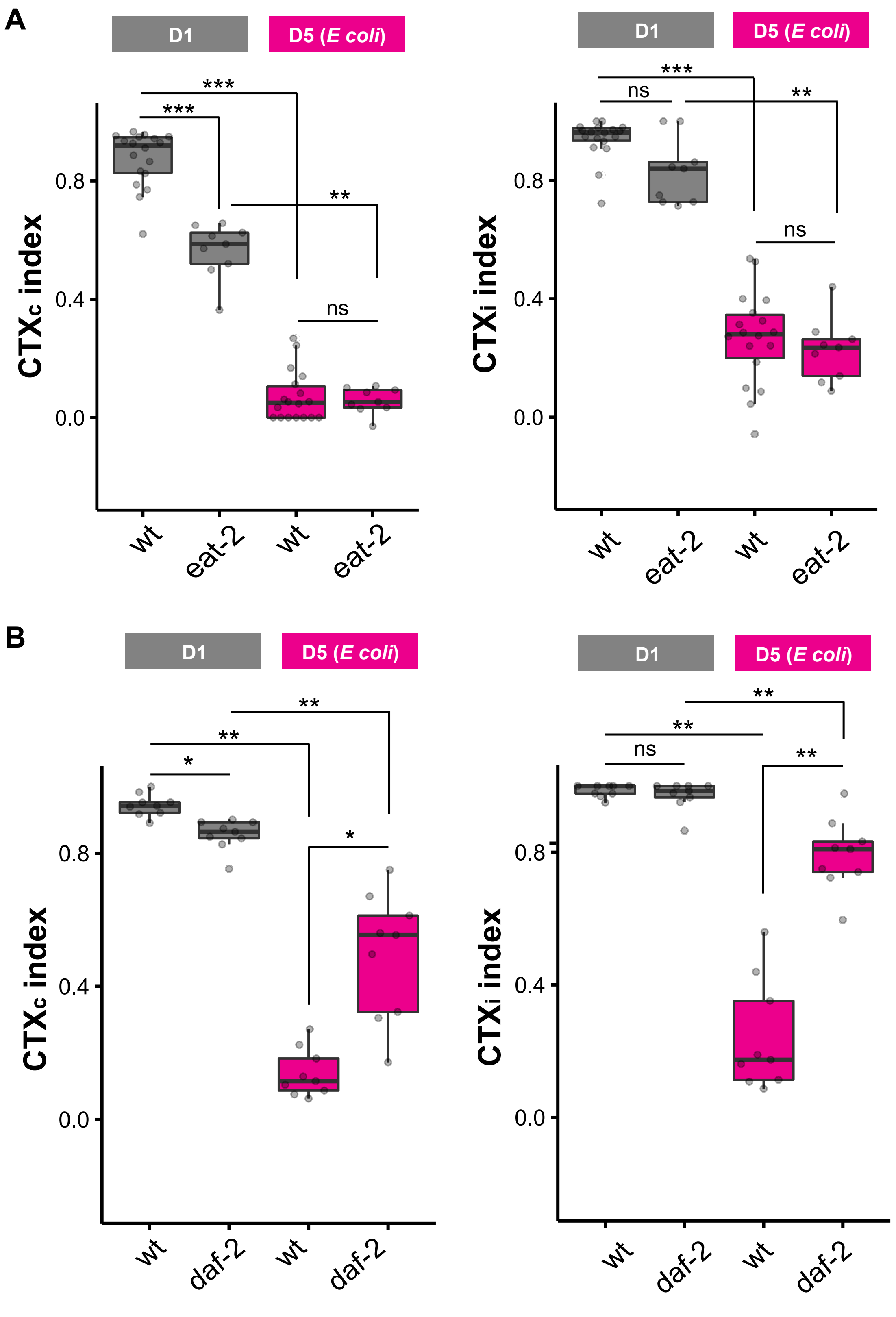

Supplementary Figure 7. Age-dependent chemotaxis decline in *eat-2* and *daf-2* mutants
Chemotaxis indices of D1 and *E. coli*-fed aged animals of *eat-2(ad1116)* and *daf-2(e1370)* mutants toward 0.1% diacetyl. Statistical difference was determined using the Kruskal–Wallis and Steel post-hoc tests for indicated pairs. ns, $p>0.05$; $^*p<0.05$; $^{**}p<0.01$; $^{***}p<0.001$.

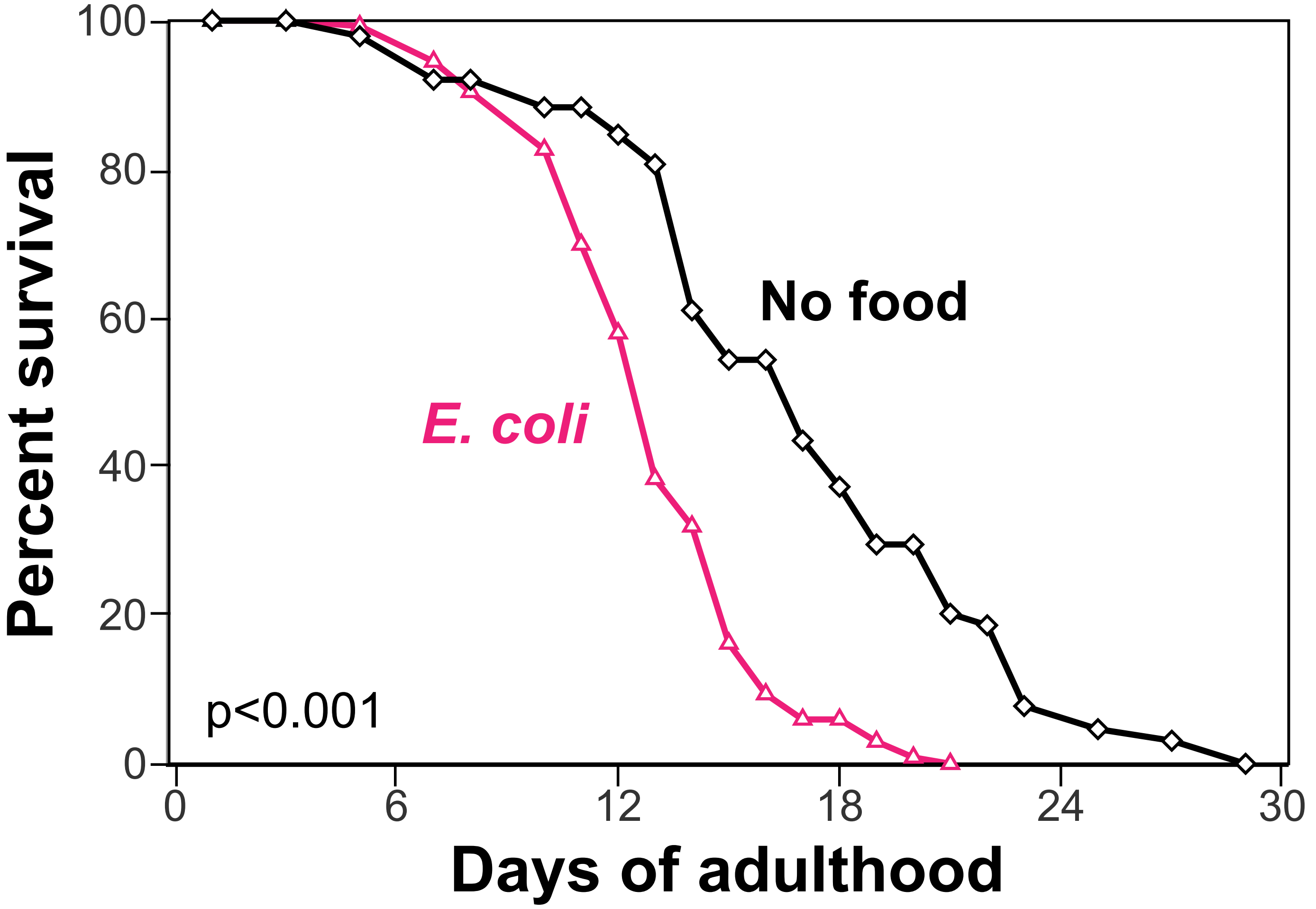

Supplementary Figure 8. The lifespan of *E. coli*-fed and food-deprived animals
The Kaplan-Meier curve for the lifespan. All the animals were cultivated with *E. coli* until D1 and transferred to plates with or without *E. coli*. Three hundred animals were tested under each condition. The Log-rank test was used to obtain the p-value.

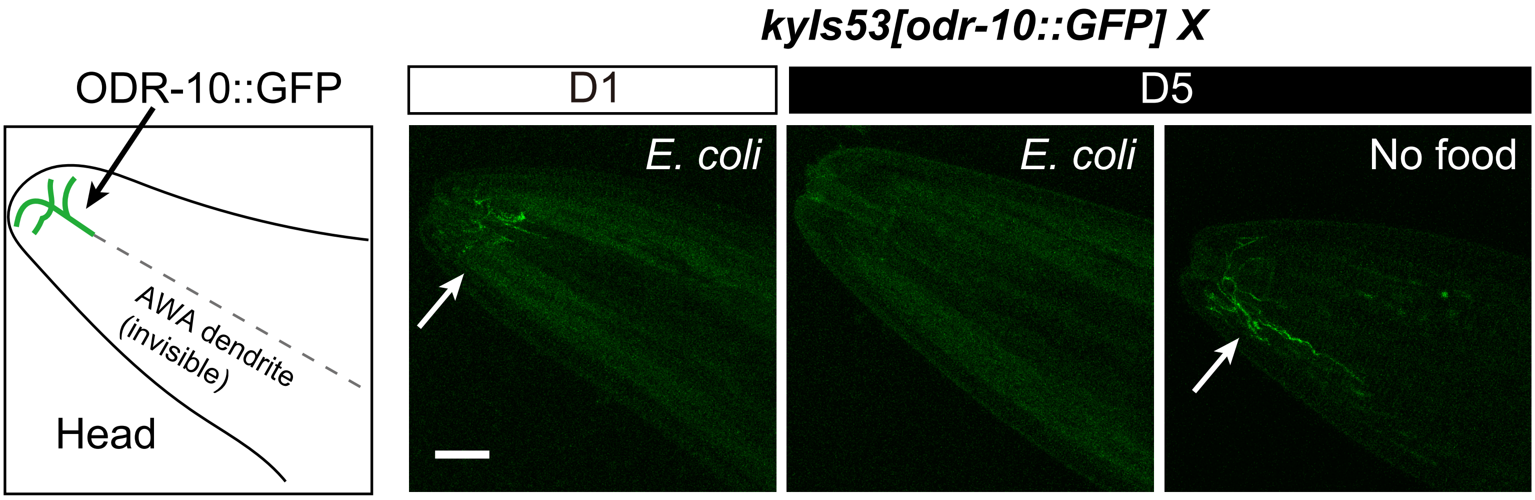

Supplementary Figure 9. Food deprivation maintains ODR-10 expression

Schematic of the head of an animal and representative images of the *odr-10* translational reporter (*kyIs53 [odr-10::GFP]*) in D1, *E. coli*-fed D5, and D5 food-deprived animals in the wild-type background. ODR-10::GFP was observed at the tip of the AWA dendrite. The scale bar indicates 10 µm.

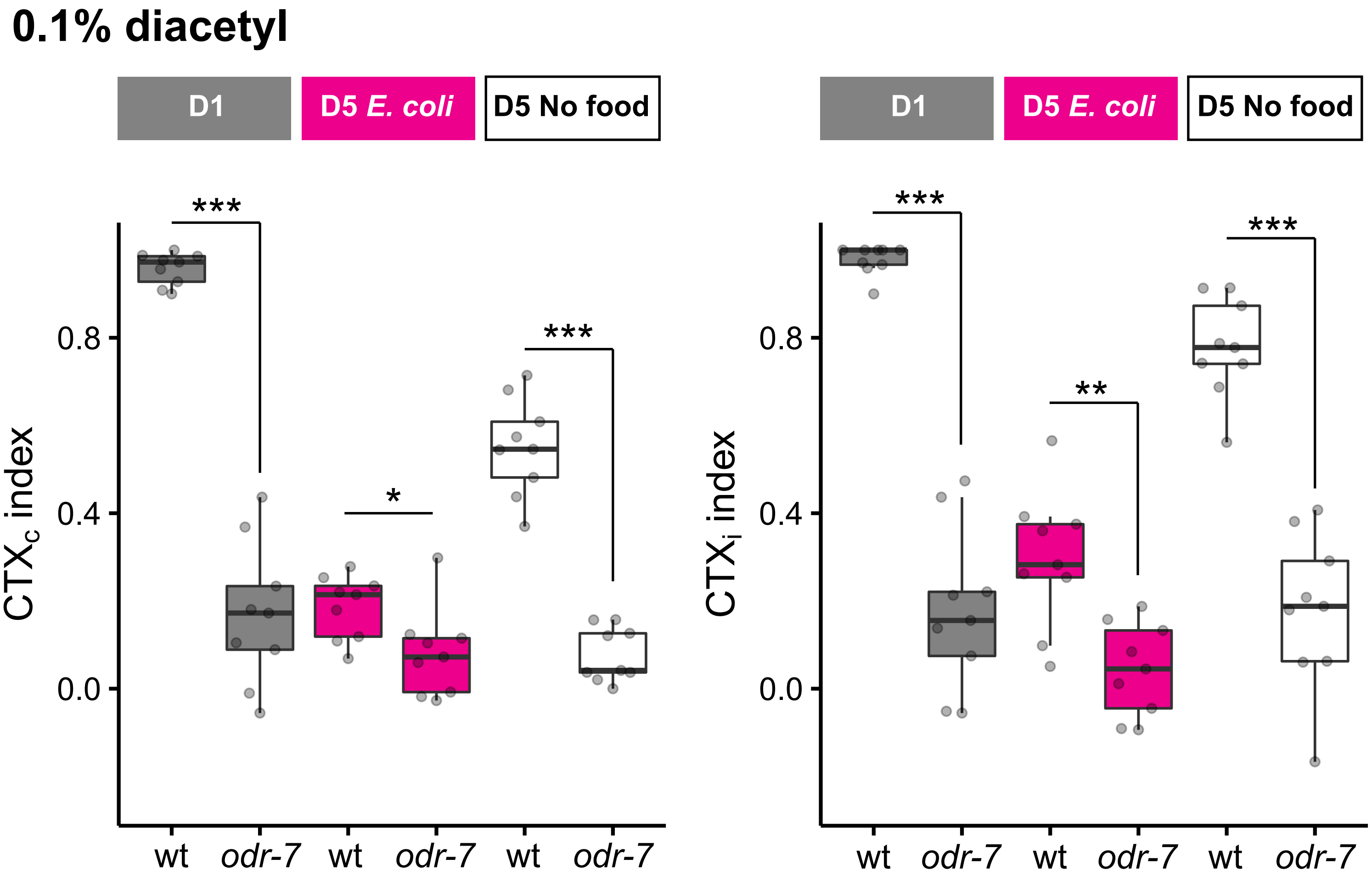

Supplementary Figure 10. *odr-7* is required for the chemotaxis of food-deprived aged animals
Chemotaxis indices of D1, D5 *E. coli*-fed, and D5 food-deprived animals with 0.1% diacetyl in the wild type and *odr-7 (ky4)*. Statistical significance was determined by the Mann-Whitney U test. *p<0.05; **p<0.01; ***p<0.001.
